# Supplementary figures and images for: CAR/CXCR5-T cell immunotherapy is safe and potentially efficacious in promoting sustained remission of SIV infection
Source: PLoS Pathog. 2022 Feb 7;18(2):e1009831. doi: 10.1371/journal.ppat.1009831 (PMC8853520; doi:10.1371/journal.ppat.1009831)

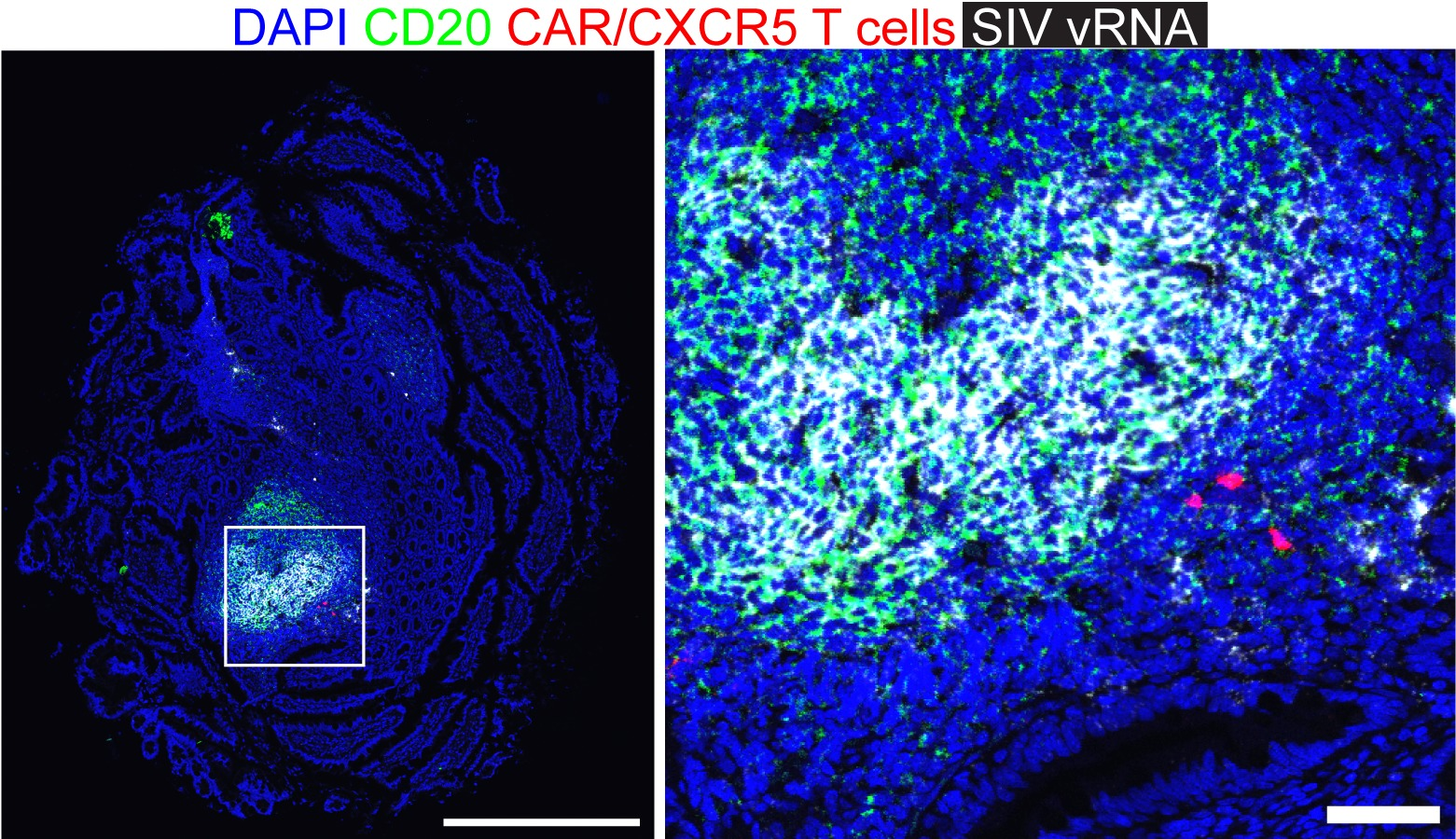

Supplement: S1 Fig — Representative image of ileum tissue from R14025 (T0), showing CAR/CXCR5-T cells (red) and SIV vRNA (white) cells detected by RNAScope ISH. The right panel is an enlargement from the left panel showing CAR/CXCR5 T cells and SIV vRNA+ cells in a lymphoid aggregate that is likely a Peyer’s patch, delineated by anti-CD20 staining (green). Scale bar is 500 μm for the left panel and 50 μm for the right panel. (TIF) [file ppat.1009831.s001.tif]

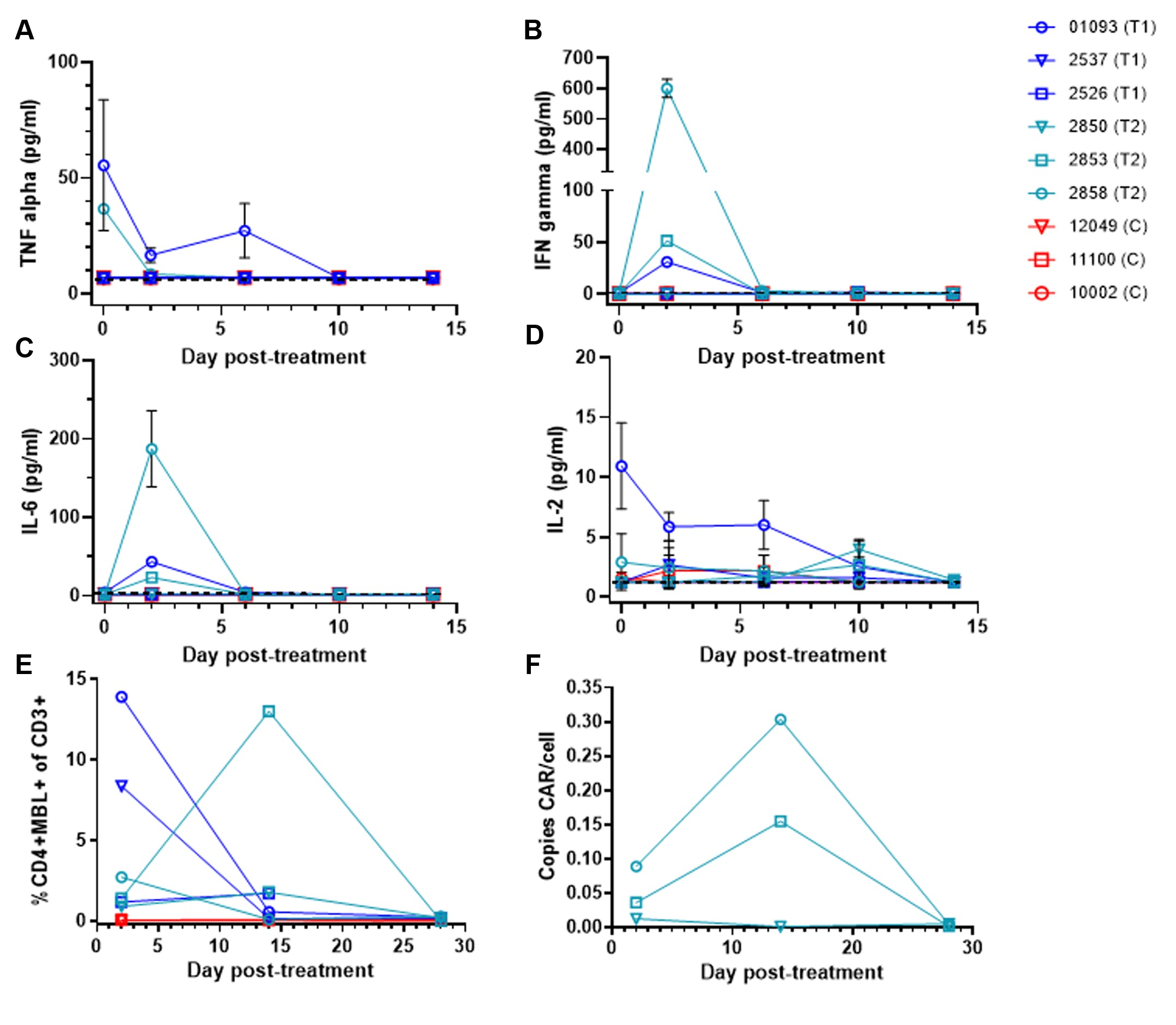

Supplement: S2 Fig — Serum samples from all treated and control animals were analyzed for post-infusion production of cytokines using a non-human primate (NHP)-specific (A) tumor necrosis factor (TNF) alpha, (B) interferon (IFN) gamma, (C) interleukin (IL)-6 and (D) IL-2 multiplex Luminex assay. Each point represents the average of two determinations with error bars representing the standard deviation. The limit of detection for each assay is indicated by the dashed line. Most determinations were below the limit of detection. Lung accumulation of CAR T cells was determined by analysis of bronchoalveolar lavage (BAL) samples. Cells were isolated from BAL and analyzed for (E) the percentage of CD4-MBL CAR+ cells in the CD3+ T population, for all treated and control animals, by flow cytometry or (F), for T2 animals only, the number of copies of CAR/cell in the total cell population by quantitative real-time PCR. (TIF) [file ppat.1009831.s002.tif]

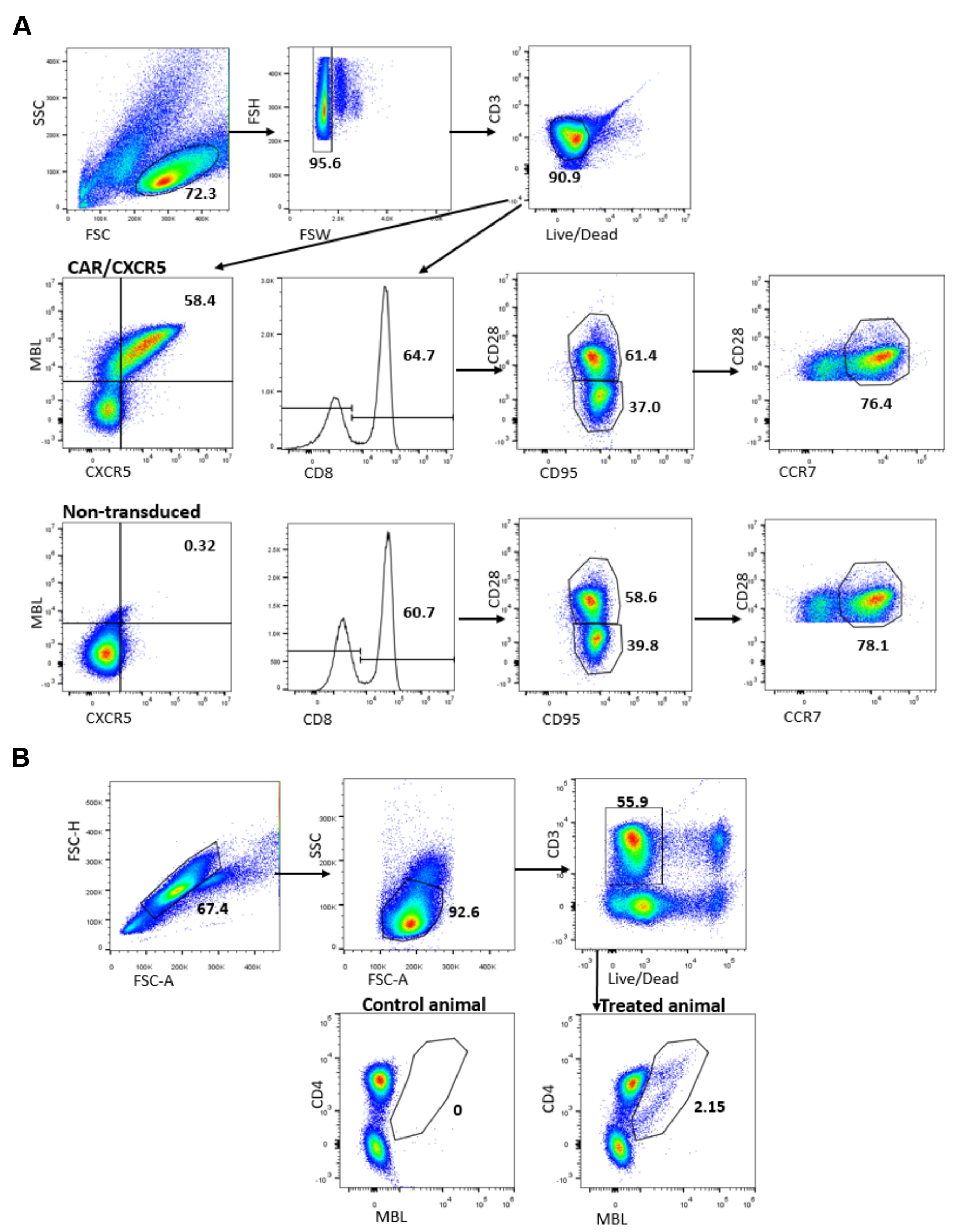

Supplement: S3 Fig — 1–2 × 10^6^ cells were stained with the antibodies listed in the Flow Cytometry section of Materials and Methods. (A) The gating strategy for determination of co-expression of CAR (MBL) and CXCR5 in the infused T cell product. The CD8+ population was used to determine central memory phenotype (CD28+CD95+) and CCR7 expression. Plots presented are from transduced cells infused into Rh2858 and from mock transduced cells from the same animal. (B) The gating strategy used to determine the percentage of infusion cells in PBMC in cells collected at multiple time-points post-infusion. Plots presented are from PBMC from a treated (R2858) and a control (R12049) animal. (TIF) [file ppat.1009831.s003.tif]

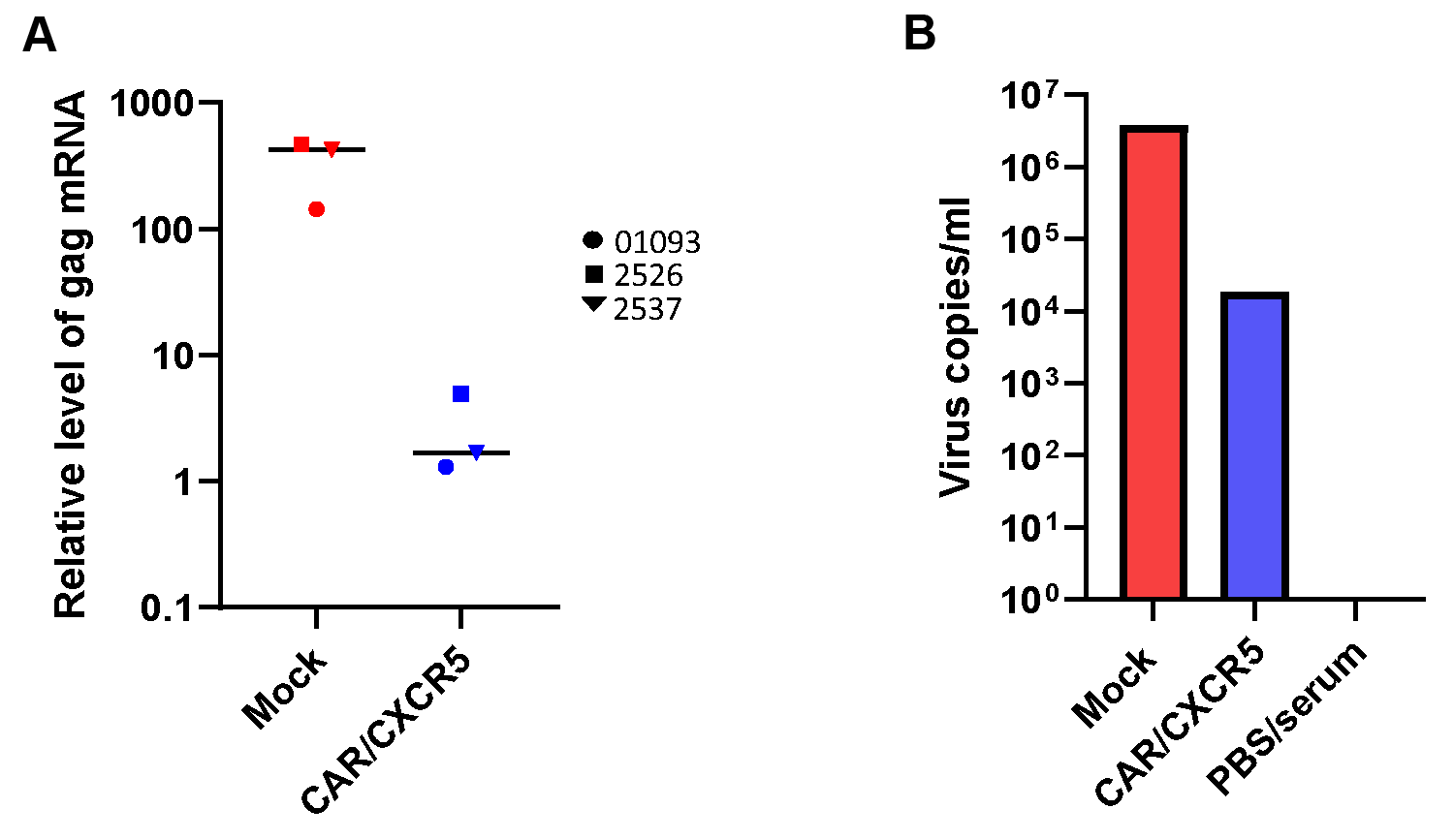

Supplement: S4 Fig — (A) Amount of gag mRNA relative to the housekeeping gene beta-actin in cell pellets using reverse transcription (RT) polymerase chain reaction (PCR). Infusion cells from the T1 animals (CAR/CXCR5) and mock transduced cells from the same animals (Mock) are presented. The bar represents the median. (B) Virus copies/ml in the supernatant of the infusion cell product for R2526 (CAR/CXCR5) as compared to the supernatant from mock transduced cells from the same animal (Mock) and the PBS/10% autologous serum used to resuspend the cells prior to transport (PBS/serum). Viral loads of the supernatant were determined by measurement of gag mRNA by reverse transcription (RT) polymerase chain reaction (PCR). (TIF) [file ppat.1009831.s004.tif]

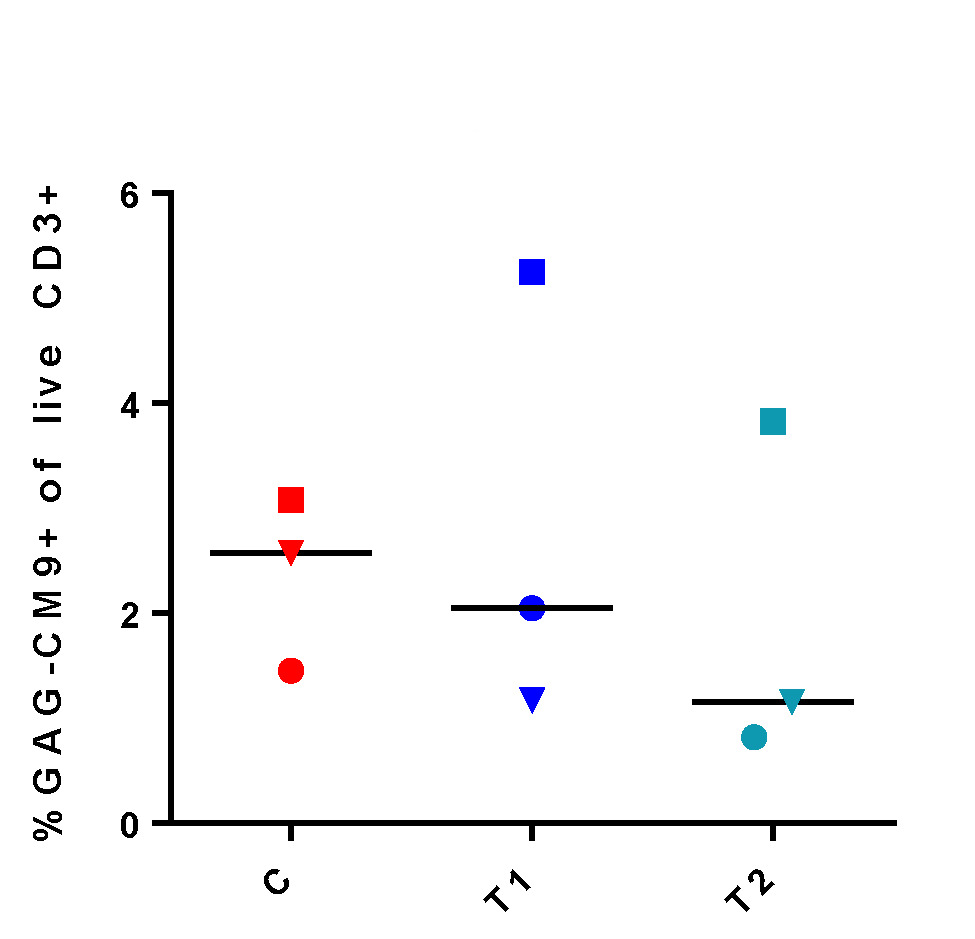

Supplement: S5 Fig — PBMC, collected on d28 from animals in each of the three groups, were stained for Gag CM9 and analyzed by flow cytometry as described in Materials and Methods. The bar represents the median. (TIF) [file ppat.1009831.s005.tif]

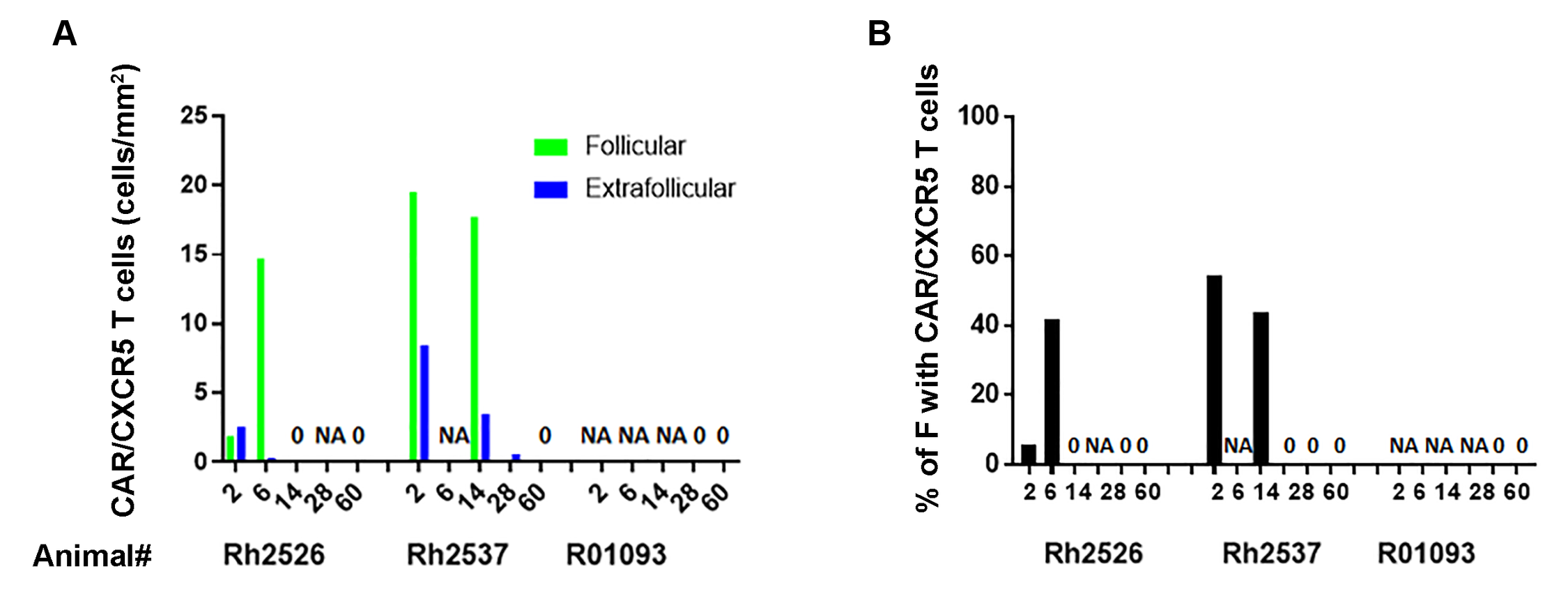

Supplement: S6 Fig — CAR/CXCR5-T cells successfully homed to the B cell follicles and persisted for up to 28 DPT in SIV-infected ART-suppressed/released animals. (A) Levels of CAR/CXCR5-T cells over time after infusion in F (green) and EF areas (blue) of LN. (B) Percentage of follicles that contained CAR/CXCR5-T cells over time post-infusion. Samples not available are marked NA. (TIF) [file ppat.1009831.s006.tif]

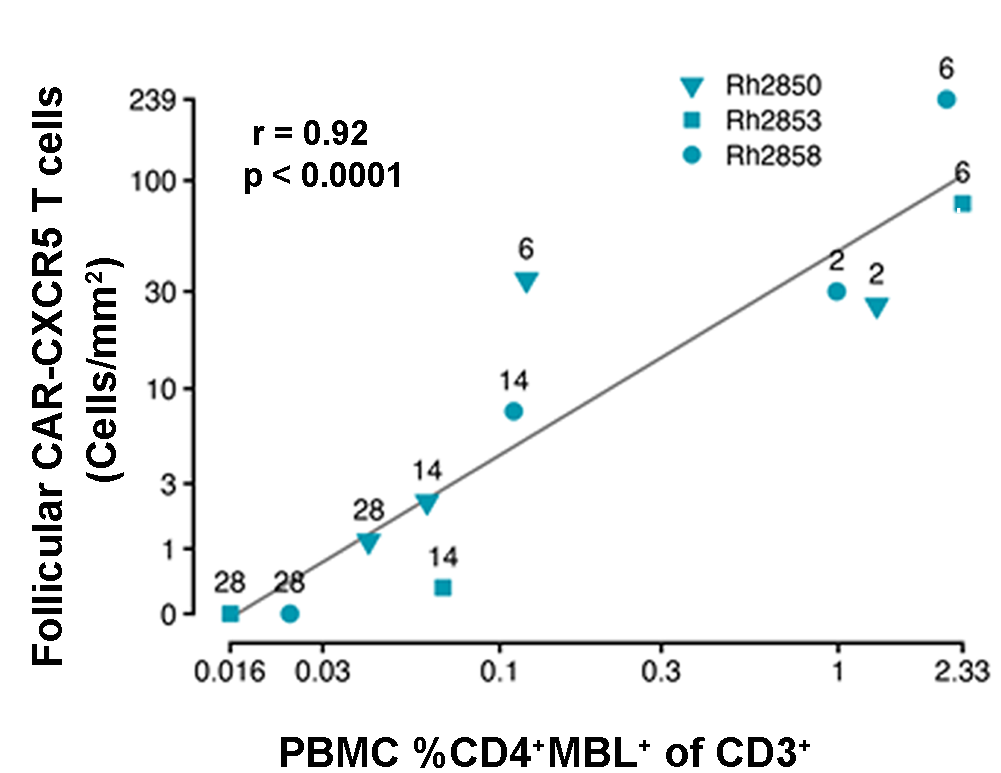

Supplement: S7 Fig — Correlation between follicular CAR/CXCR5-T cells/mm2 by RNAScope and CD4-MBL+ PBMC by flow cytometry. Association was tested using Spearman’s correlation. Scales are log (value+1) on the y-axis and log (value) on the x-axis; labels use the original units. The line represents the fitted regression. Points are labeled by days post-treatment (2, 6, 14, and 28) with a unique shape for each animal. (TIF) [file ppat.1009831.s007.tif]
