# Supplementary material for: CAR/CXCR5-T cell immunotherapy is safe and potentially efficacious in promoting sustained remission of SIV infection
Source: PLoS Pathog. 2022 Feb 7;18(2):e1009831. doi: 10.1371/journal.ppat.1009831 (PMC8853520; doi:10.1371/journal.ppat.1009831)
Supplement: Attachment — Submitted filename: Response to reviewers.pdf [file ppat.1009831.s008.pdf]

November 5, 2021

Dear Drs. Duoek, Hope, Haldar and Malim,

We would like to thank the reviewers for their thoughtful evaluation of our manuscript "CAR/CXCR5-T cell immunotherapy is safe and potentially efficacious in promoting sustained remission of SIV infection". We appreciate that the reviewers thought that "it was a well-designed and important study applying cutting-edge imaging assays to promote a promising approach to HIV cure" and that "the rationale, strategy and model system in this study can potentially provide unique insight into a CAR based approach to specifically target a persistently infected reservoir." We recognize that there were certain limitations to the study and have adjusted our language accordingly. We have added pertinent information and data as requested by both reviewers. Each point is addressed below.

We have also amended our manuscript to add additional data that has been obtained since our original submission. These include adding a supplemental figure showing the detection of CAR/CXCR5 T cells in lymphoid aggregates ileum tissue, and noting that we identified one CAR/CXCR5+ cell that is vRNA+ (out of 689 cells examined).

Major Issues:

Reviewer 1

- **In Figures 1A, 4A-B, and elsewhere, the difference between CTV and CAR/CXCR5 RNAscope labeling should be clarified. CTV will label the entire infused cell product, not just the 55.0-79.4% of cells that have been modified with the CAR/CXCR5 vector. It would be more accurate to refer to data using CTV as "the infused T cell product," rather than CAR/CXCR5-T cells, and CTV and CAR/CXCR5-T RNAscope labeling should preferably be shown in different colors, to avoid confusion.**
  That is a very good point. We have done the following to address this concern:
  1. Changed the language to indicate "infused T cell product" when referring to the chronically infected animal that received CTV labeled cells.
  2. Separated Figure 1 into 2 figures to more easily distinguish the cells detected by CTV from those detected with RNAscope.
  3. Pseudocolored the CTV cells magenta to help distinguish the CTV-labeled cells from the cells detected with the RNAscope in situ hybridization (which remain red).

- **What was the frequency of CAR+ (and CTV+) cells in the blood in the pilot animal in Figure 1?**
  Immediately after infusion, the frequency of CTV+ cells was 0.17% of the total lymphocyte population in the blood. That level dropped to 0.055% at necropsy. That information has been added to the manuscript.

- **The authors state from their previous work that "the average ratio of in vivo effector (virus-specific CD8+ T cells) to target (virus-infected cells) cells is over 40-fold lower in follicular (F) compartments compared to extrafollicular (EF) compartments of secondary lymphoid tissues" (lines 68-70). Can this ratio be quantified in the current manuscript? How does it compare to the percentage of follicles with CAR+ T cells (Figure 5D) and the percent of SIV vRNA+ cells in direct contact with CAR/CXCR5-T cells (Figure 1B)?**

We have clarified the text to say that we see clear evidence for in vivo replication of the infused cells and have removed assertions regarding "expansion without addition of exogenous antigen."

Reviewer 2

- **A key issue with every aspect of the study in addressing their hypothesis is the lack of proper controls, ideally with animals that received non-transduced or control vector modified cells. The control animals, from all appearances, were not treated with an infused cell product. Due to this, and the reported fact that approximately 40% of the cells that were injected into the treated animals were not expressing, or were not transduced, with the CAR/CXCR5 vector, it is difficult to determine if greater levels of CAR expressing cells were infiltrating the follicles due to CXCR5 co-expression. Since the entire cell product was labeled with the fluorescent Cell Trace Violet (CTV), it is not clear whether the CAR/CXCXR5 vector modified cells were behaving in any way different than unmodified cells or infused cells in a control animal. It also is not clear, particularly in animals that received CAR/CXCR5 modification after SIV infection, whether the cells found in the follicles are endogenous SIV-specific T cells from the infused CTV-labeled fraction or are other cells that migrated there. There is some RNAscope in situ hybridization presented that shows localization of CAR/CXCR5 modified cells in and around the follicle, but it is not clear that the presence of vector expressing CXCR5 increases this. In addition, the levels of CAR/CXCR5 modified cells versus unmodified cell populations at these anatomical sites are not clear. The inclusion of a control, optimally animals receiving cells modified with a control vector, or another manner of analyzing the present data would strengthen their conclusions that the CXCR5 component on the cells was facilitating greater follicular localization.**

  Unfortunately, we were not able to include an arm of the study with animals infused with non-transduced cells or CAR-only cells. However, previous work, as cited in the paper, has shown that virus-specific CD8 T cells do not generally accumulate to high levels in lymphoid follicles [2–5]. This is likely due to only a small percentage of endogenous virus-specific CD8 T cells expressing the B cell follicle homing molecule (CXCR5)[3]. Additionally, a previous study by Ayala et al. demonstrated, in rhesus macaques, that CD8 T cells engineered to express CXCR5 accumulated in follicles while unmodified CD8 T cells did not accumulate in follicles, but rather accumulated in extrafollicular T cell zones[6]. This study demonstrated that CXCR5 expression induces the accumulation in lymphoid follicles, and that cells without CXCR5 do not accumulate in follicles. Further, we demonstrated, in an ex vivo tissue migration assay, that cells expressing CAR/CXCR5 preferentially accumulated in lymphoid follicles while cells expressing CAR alone did not[7]. These studies demonstrate that CXCR5 expression on lymphocytes is sufficient to induce their specific migration and accumulation in follicles.

  While the CTV-labeled do not allow the discernment of the transduced and non-transduced populations, subsequent studies using RNAScope in situ hybridization combined with immunofluorescence, specifically detected only CAR/CXCR5 T cells allowing us to detect only the transduced cell population. CTV-labeled cells showed increased concentrations in extrafollicular areas compared to RNAscope labeled CAR/CXCR5 T cells suggesting that the non-transduced cells were largely located in extrafollicular areas.

Addressing the concerns that endogenous SIV-specific T cells are exerting an effect, we note that the cells transduced for the T2 group were collected prior to SIV infection making it unlikely that many if any were endogenous SIV-specific T cells. Additionally, we did in situ tetramer staining to stain virus-specific T cells in the biopsies from the T1 treated animals and found few to no gag/CM9+/CTV+ cells in the lymph nodes indicating that very few if any of the infused T cell product were immunodominant gag/CM9+ T cells. Furthermore, flow cytometry of the CAR/CXCR5 T cells in the blood post-infusion reveals that none of the CAR cells detected were gag/CM9+.

We have amended the manuscript to clarify these points.

- **The inclusion of a control along with or in the "initial" animal data shown in Figure 1 would strengthen these results and make them more meaningful. It is not clear if unmodified cells would anatomically distribute this way as well.**
While we did not have a control animal with the first treated animal, we did include 3 control animals which did not receive therapeutic cells that were used as a negative control for CTV-labeled infused T cell product detection. Figure 1 (previously figure 1a) shows distribution of CTV-labeled infused T cells post-infusion in lymphoid and non-lymphoid tissues. Importantly, we did not detect accumulations of CTV+ cells in the non-lymphoid tissue sections examined from both the treated and untreated negative control animals. These data suggest that neither the transduced nor the non-transduced CTV-labeled cells accumulated in the non-lymphoid tissues examined.

Importantly, Figure 2 (previously Figure 1b) utilized RNAsope in situ hybridization using a probe that specifically detects the CAR/CXCR5 transduced T cells. The RNAscope ISH combined with immunofluorescence confirmed that the CAR/CXCR5 T cells accumulated in the follicular areas of the lymphoid tissues. Additionally, as mentioned above, Ayala et al., have previously demonstrated that unmodified T cells (cells that do not express CXCR5), do not accumulate in follicles, but rather accumulate in extrafollicular T cell zone areas. We have clarified these points in the text of the revised manuscript.

- **The viral load data described in Figure 3 appear to indicate possible effects of CAR/CXCR5 modified infused cells in 4 of 6 animals. However, there is natural decrease in the untreated control animals to low levels at the final time points as well. Since this control did not receive infused cells, the significance of this is not clear (other groups have demonstrated that untreated cell infusions can affect viral loads). In addition, without matched controls for the extended time period in the T2 animals shown in Figure 3c, the significance of the presence of low viral loads at these later time points is not clear. In the absence of detection of CAR/CXCR5 expressing cells, is this a natural phenomenon?**

We acknowledge in the manuscript that there were no control animals that were maintained for up to 10 months post-ATI. The animals in T1 were maintained for 2-3 months as originally planned and were used as historical controls for T2. Historic controls from the literature show that it is not unusual for VLs in SIVmac infected A01 animals to drop to low levels after ART release. However, this drop is temporary, and it is rare for animals to control infection for 10 months [8–10]. While we cannot absolutely rule out the possibility of a natural decrease in viral

load, historical data supports the conclusion that it is likely that the control animals, if left in the study longer, would have shown increases in viremia.

While it is true that infusion of autologous ex vivo expanded CD8 T cells may have led to improved clinical outcomes, it is unclear whether there was any impact on viral loads[11]. Infusion of autologous HIV-specific CD8 T cells showed either no impact on viral loads[12], or there was a decrease in viral loads in a subset of subjects during the first two weeks after infusion, but that reduction was not sustained [13]. In addition, infusion of virus-specific T cells into SIV infected rhesus macaques did not impact viral loads[14].

It is indeed perplexing that the T2 animals maintained control of viremia for 10 months, long after the CAR/CXCR5 T cells became undetectable. We theorize that the CAR/CXCR5 T cells provided early control and clearance of the recrudescent SIV-producing cells, thus providing time for the endogenous CTL response to be stimulated and re-emerge to maintain control. In addition, the CAR/CXCR5 T cell clearing of emerging virus producing cells in follicles may have led to decreased viral production over time. These points are clarified in the revised manuscript.

Minor issues

<u>Reviewer 1</u>

- **Were all animals A*01+? If not, could MHC haplotype contribute to virus control in any of the animals?**
  All animals were A01. We have clarified this in the results section of the manuscript and it is described in the materials and methods section as well.

- **Despite statements in lines 119-120, the animals in cohorts C, T1, and T2 do not appear to be particularly well-matched in terms of age, sex, or weight.**
  We have removed this statement from the manuscript.

- **It would be helpful to expand Figure 2 to include schematics of what tissues were collected from each animal at which time points.**
  The timeline figure (Figure 2) has been amended to indicate sample collections (Figure 3 in the revised manuscript).

- **Figure 3 appears to show plasma viral loads, which makes the reference in the Figure 3 legend to a beta actin housekeeping gene and use of cell pellets confusing.**
  Thank you for finding this error in the figure legend. It has been corrected to read "Viral loads were determined by measurement of gag mRNA in plasma using reverse transcription (RT) polymerase chain reaction (PCR)."

- **What does the staining pattern shown in Figure S2 look like in animal(s) from the control cohort, i.e. is this flow cytometry assay specific for CAR+ cells?**
  We have revised Supplementary figure S2 (now S3) to show the staining patterns for mock transduced cells from the same treatment animal to more clearly indicate the gating strategy and show specificity of the gating for CAR/CXCR5 during cell production. We have also added the

gating strategy for examination of PBMC after infusion using flow cytometry to stain CAR T cells in treated and control animals. No CAR T cells were detected in control animals.

In Figures 4-5, are data available from cohort T1?
We have incomplete data available from cohort T1 due to some lymphoid biopsy samples not being available. The data that we do have has been added as supplementary figure 6.

- **The statement in the discussion, "Interestingly, we detected no SIV vRNA in CAR/CXCR5 cells in the examined sections." (lines 325-326) should be tempered, indicating that only imaging-based assays were applied. Alternatively, flow- and PCR-based assays to confirm that CAR+ cells are not infected in vivo would significantly strengthen this assertion.**
  We have tempered the statement acknowledging that only imaging assays were conducted.

Reviewer 2

- **The methodology involved in the overall approach is not clear. A better description of key elements of this, such as greater details of the CAR/CXCR5 vector, cell processing methodologies/cell activation techniques, and the inclusion or absence of conditioning (such as lymphodepletion) regimens would strengthen this manuscript. Some reference to these is provided, but a clearer description and inclusion of these in the text would strengthen this manuscript.**
  As suggested we have added a better description of the key elements of our study methods. Specifically, to the animal paragraph, we added a statement that the animals did not undergo a lymphodepletion regimen prior to infusion of CAR/CXCR5 T cells. To the cell production paragraph we added more details concerning the CAR/CXCR5 construct, viral vector production, cell activation and cell processing.

We thank the editors and the reviewers for the opportunity to make these improvements to the manuscript. This revised manuscript presents important findings in a potential treatment for HIV with a CAR-based immunotherapy and we appreciate your consideration of the article for publication in Plos Pathogens.

Sincerely,

Mary Pampusch

Mary Pampusch

References:

1. Mothé BR, Horton H, Carter DK, Allen TM, Liebl ME, Skinner P, et al. Dominance of CD8 responses specific for epitopes bound by a single major histocompatibility complex class I molecule during the acute phase of viral infection. J Virol. 2002;76. doi:10.1128/JVI.76.2.875-884.2002

2. Connick E, Mattila T, Folkvord JM, Schlichtemeier R, Meditz AL, Graham Ray M, et al. CTL Fail to Accumulate at Sites of HIV-1 Replication in Lymphoid Tissue. The Journal of Immunology. 2007. pp. 6975–6983. doi:10.4049/jimmunol.178.11.6975

3. Connick E, Folkvord JM, Lind KT, Rakasz EG, Miles B, Wilson NA, et al. Compartmentalization of simian immunodeficiency virus replication within secondary lymphoid tissues of rhesus macaques is linked to disease stage and inversely related to localization of virus-specific CTL. J Immunol. 2014;193: 5613–5625. doi:10.4049/jimmunol.1401161

4. Tjernlund A, Zhu J, Laing K, Diem K, McDonald D, Vazquez J, et al. In situ detection of Gag-specific CD8+ cells in the GI tract of SIV infected Rhesus macaques. Retrovirology. 2010;7: 12. doi:10.1186/1742-4690-7-12

5. Sasikala-Appukuttan AK, Kim HO, Kinzel NJ, Hong JJ, Smith AJ, Wagstaff R, et al. Location and dynamics of the immunodominant CD8 T cell response to SIVΔnef immunization and SIVmac251 vaginal challenge. PLoS One. 2013;8. doi:10.1371/journal.pone.0081623

6. Ayala VI, Deleage C, Trivett MT, Jain S, Coren LV, Breed MW, et al. CXCR5 Dependent Entry of CD8 T Cells into Rhesus Macaque B-Cell Follicles Achieved Through T-Cell Engineering. J Virol. 2017;2: JVI.02507–16. doi:10.1128/JVI.02507-16

7. Haran KP, Hajduczki A, Pampusch MS, Mwakalundwa G, Vargas-Inchaustegui DA, Rakasz EG, et al. Simian Immunodeficiency Virus (SIV)-Specific Chimeric Antigen Receptor-T Cells Engineered to Target B Cell Follicles and Suppress SIV Replication. Front Immunol. 2018;9: 492. doi:10.3389/fimmu.2018.00492

8. Borducchi EN, Cabral C, Stephenson KE, Liu J, Abbink P, Ng'ang'a D, et al. Ad26/MVA therapeutic vaccination with TLR7 stimulation in SIV-infected rhesus monkeys. Nature. 2016;540: 284–287. doi:10.1038/nature20583

9. Strongin Z, Micci L, Fromentin R, Harper J, McBrien J, Ryan E, et al. Virologic and Immunologic Features of Simian Immunodeficiency Virus Control Post-ART Interruption in Rhesus Macaques. J Virol. 2020;94. doi:10.1128/JVI.00338-20

10. Lim S-Y, Osuna CE, Hraber PT, Hesselgesser J, Gerold JM, Barnes TL, et al. TLR7 agonists induce transient viremia and reduce the viral reservoir in SIV-infected rhesus macaques on antiretroviral therapy. Sci Transl Med. 2018;10.

doi:10.1126/scitranslmed.aao4521

11. Ho M, Armstrong J, McMahon D, Pazin G, Huang XL, Rinaldo C, et al. A phase 1 study of adoptive transfer of autologous CD8+ T lymphocytes in patients with acquired immunodeficiency syndrome (AIDS)-related complex or AIDS. Blood. 1993;81: 2093–2101. Available: https://www.ncbi.nlm.nih.gov/pubmed/8471768

12. Tan R, Xu X, Ogg GS, Hansasuta P, Dong T, Rostron T, et al. Rapid death of adoptively transferred T cells in acquired immunodeficiency syndrome. Blood. 1999;93: 1506–1510. Available: https://www.ncbi.nlm.nih.gov/pubmed/10029578

13. Lieberman J, Skolnik PR, Parkerson GR, Fabry JA, Landry B, Bethel J, et al. Safety of autologous, ex vivo-expanded human immunodeficiency virus (HIV)-specific cytotoxic T-lymphocyte infusion in HIV-infected patients. Blood. 1997;90: 2196–2206. Available: https://www.ncbi.nlm.nih.gov/pubmed/9310470

14. Ayala VI, Trivett MT, Barsov EV, Jain S, Piatak M, Trubey CM, et al. Adoptive Transfer of Engineered Rhesus Simian Immunodeficiency Virus-Specific CD8 T Cells Reduces the Number of Transmitted/Founder Viruses Established in Rhesus Macaques. Journal of Virology. 2016. pp. 9942–9952. doi:10.1128/jvi.01522-16
