# Supplementary material for: CAR/CXCR5-T cell immunotherapy is safe and potentially efficacious in promoting sustained remission of SIV infection
Source: PLoS Pathog. 2022 Feb 7;18(2):e1009831. doi: 10.1371/journal.ppat.1009831 (PMC8853520; doi:10.1371/journal.ppat.1009831)
Supplement: Attachment — Submitted filename: response to reviewers_011322.pdf [file ppat.1009831.s009.pdf]

January 13, 2022

Dear Drs. Duoek, Hope, Haldar and Malim,

We would like to thank the reviewers for thoughtfully examining our revised manuscript, "CAR/CXCR5-T cell immunotherapy is safe and potentially efficacious in promoting sustained remission of SIV infection". We appreciate that they thought that we "presented a revised manuscript containing important data updates" and that we submitted "a significantly revised manuscript that sufficiently addresses previous concerns".

In order to address the minor issues raised by Reviewer 1, we have made revisions to the Materials and Methods and Results sections. We have updated Table 3 and Figure 6c as well as the data presented describing the first treated animal, reflecting additional analyses performed and data generated since our last submission.

Minor issues, Reviewer 1:
Additional imaging-based follicular E:T ratio data is a key aspect of the revised manuscript, but no methodology is presented. Important details to include in the manuscript include:

i) how these values were calculated
Effector SIV-specific CAR/CXCR5 T cells to SIV RNA+ target cell (E: T) ratios in secondary lymphoid tissues were calculated by dividing the levels of CAR/CXCR5 T cells/mm^2 by the level of SIV RNA+ cells/mm^2 within a specific tissue area. For these analyses, for each animal we examined 2 or 3 tissue sections, with at least 6 follicles (range 6-9) examined per animal. To determine the range of E:T ratios when the denominator was 0, we used the value of the numerator. When the numerator was zero, 1 was used as the numerator. For determination of the distance between E and T cells in R14025, 3-9 follicles were examined from each of 3 spleen sections and the distance of follicular vRNA+ cells to the nearest CAR/CXCR5+ cell was measured. For the T2 animals, all follicular vRNA+ cells were evaluated in 2 sections (8-28 follicles/section) for each animal. The methodology has been added to the Materials and Methods section of the manuscript.

ii) the maximum distance between a given CAR RNA+ effector and viral RNA+ target

The distance between the observed SIV+ cells and the nearest CAR/CXCR5 T cells has been measured for the chronically infected ART naive animal, R14025 as well as for the three animals in the T2 study. For the ART naive animal, where 119 follicular SIV vRNA+ cells were measured, the median distance between E and T was 86 µm with a range of 0-441 µm. We had already previously noted the number of vRNA+ cells in direct contact with CAR/CXCR5 cells. For the T2 animals, the median distance between E and T in follicles was 60 µm with a range of 0-170 µm. The distance information is presented in the Results section of the manuscript.

iii) whether this distance would be expected to facilitate direct target killing
CAR/CXCR5 T cells need to migrate towards the SIV RNA+ target cell and directly contact target cells to provide death-inducing signals via a cytotoxic immunological synapse. Some CAR/CXCR5 T cells were found in direct contact with virally infected cells. The vRNA+ cell furthest from a CAR/CXCR5 T cell was 441 um away. The tissue sections analyzed captured a snapshot of the location of the cells at the time of biopsy. It is important to note that, in vivo, T cells are moving at an average speed of 10 μm per minute in secondary lymphoid tissues [1]. In theory, given this rate of movement, our data suggests that the CAR/CXCR5 T cells were close enough to each of the vRNA+ evaluated to contact and kill them in a relatively short period of time. We have added this consideration to the Discussion section of the manuscript.

iv) possible limitations to calculating E:T ratios using RNA signal, e.g. due to CAR RNA+ and/or viral RNA+ cells that aren't expressing CAR/virus proteins.

We acknowledge that a limitation of our calculated E:T ratios is the assumption that the cells detected by RNAscope were for the most part expressing CAR/virus protein. Since most SIV vRNA+ cells including those expressing low levels of vRNA express viral protein [2], we are fairly confident that most if not all of the SIV vRNA+ cells detected in our studies were expressing viral proteins. While we did not directly evaluate CAR/CXCR5 protein expression in the RNAscope studies, we saw, by flow cytometry, that the cells expressed CAR and CXCR5 proteins on cell surfaces when infused into the animals (Table 2, Fig S3), and know that when we detected CAR/CXCR5 T cells via RNAscope in tissues, we were at the same time able to detect cells expressing CAR and CXCR5 proteins in the blood (Fig 6e, Fig S3). Furthermore, in more recent studies not presented in this paper, we have detected cells expressing CAR/CXCR5 proteins in disaggregated lymph nodes at the same time CAR/CXCR5+ cells were detected by RNAscope. Finally, the CAR/CXCR5 T cells accumulated in follicles (Fig 6), which is rare without CXCR5 expression [3,4].

We would like to thank the editors and reviewers for examining our revised manuscript and allowing us to make further improvements. We hope that this additional data strengthens our manuscript and that you will consider it for publication in Plos Pathogens.

Sincerely,

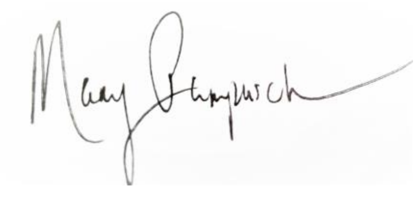

Mary Pampusch

References:

1. Katakai, T and Kinashi T. Microenvironmental Control of High-Speed Interstitial T Cell Migration in the Lymph Node Front. Immunol. 13 May 2016 https://doi.org/10.3389/fimmu.2016.00194

2. Zhang Z, Schuler T, Zupanicic M, Wietgrefe S, Staskus K et al. Sexual Transmission and Propagation of SIV and HIV in Resting and Activated CD4+ T Cells Science 1999; 286 (5448): 2273

3. Ayala VI, Deleage C, Trivett MT, Jain S, Coren LV, Breed MW, et al. CXCR5-Dependent Entry of CD8 T Cells into Rhesus Macaque B-Cell Follicles Achieved through T-Cell Engineering. J Virol. 2017;91. doi:10.1128/JVI.02507-16

4. Haran KP, Hajduczki A, Pampusch MS, Mwakalundwa G, Vargas-Inchaustegui DA, Rakasz EG, et al. Simian Immunodeficiency Virus (SIV)-Specific Chimeric Antigen Receptor-T Cells Engineered to Target B Cell Follicles and Suppress SIV Replication. Front Immunol. 2018;9: 492. doi:10.3389/fimmu.2018.00492
